# Air pollution exposure and lung function in highly exposed subjects in Beijing, China: a repeated-measure study

**DOI:** 10.1186/s12989-014-0051-7

**Published:** 2014-10-02

**Authors:** Andrea A Baccarelli, Yinan Zheng, Xiao Zhang, Dou Chang, Lei Liu, Katherine Rose Wolf, Zhou Zhang, John P McCracken, Anaité Díaz, Pier Alberto Bertazzi, Joel Schwartz, Sheng Wang, Choong-Min Kang, Petros Koutrakis, Lifang Hou

**Affiliations:** Department of Environmental Health, Harvard School of Public Health, Boston, MA USA; Institute for Public Health and Medicine, Feinberg School of Medicine, Northwestern University, Chicago, IL USA; Department of Preventive Medicine, Feinberg School of Medicine, Northwestern University, Chicago, IL USA; Department of Safety Engineering, China Institute of Industrial Health, Beijing, China; Driskill Graduate Program in Life Sciences, Feinberg School of Medicine, Northwestern University, Chicago, IL USA; Center for Health Studies, Universidad del Valle de Guatemala, Guatemala City, Guatemala; Center of Molecular and Genetic Epidemiology, Department of Clinical Sciences and Community Health, University of Milan and IRCCS Foundation Ca’ Granda, Ospedale Maggiore Policlinico, Italy; Department of Occupational and Environmental Health, Peking University Health Science Center, No. 38 Xueyuan Road, Haidian District, Beijing, 100191 China; Robert H. Lurie Comprehensive Cancer Center, Feinberg School of Medicine, Northwestern University, Chicago, IL USA

**Keywords:** Lung function, Metals, Particulate matter, Traffic exposure, FEV1, FVC

## Abstract

**Background:**

Exposure to ambient particulate matter (PM) has been associated with reduced lung function. Elemental components of PM have been suggested to have critical roles in PM toxicity, but their contribution to respiratory effects remains under-investigated. We evaluated the effects of traffic-related PM_2.5_ and its elemental components on lung function in two highly exposed groups of healthy adults in Beijing, China.

**Methods:**

The Beijing Truck Driver Air Pollution Study (BTDAS) included 60 truck drivers and 60 office workers evaluated in 2008. On two days separated by 1-2 weeks, we measured lung function at the end of the work day, personal PM_2.5_, and nine elemental components of PM_2.5_ during eight hours of work, i.e., elemental carbon (EC), potassium (K), sulfur (S), iron (Fe), silicon (Si), aluminum (Al), zinc (Zn), calcium (Ca), and titanium (Ti). We used covariate-adjusted mixed-effects models including PM_2.5_ as a covariate to estimate the percentage change in lung function associated with an inter-quartile range (IQR) exposure increase.

**Results:**

The two groups had high and overlapping exposure distributions with mean personal PM_2.5_ of 94.6 μg/m^3^ (IQR: 48.5-126.6) in office workers and 126.8 μg/m^3^ (IQR: 73.9-160.5) in truck drivers. The distributions of the nine elements showed group-specific profiles and generally higher levels in truck drivers. In all subjects combined, forced expiratory volume in 1 second (FEV1) and forced vital capacity (FVC) did not significantly correlate with PM_2.5_. However, FEV1 showed negative associations with concentrations of four elements: Si (-3.07%, 95% CI: -5.00; -1.11, IQR: 1.54), Al (-2.88%, 95% CI: -4.91; -0.81, IQR: 0.86), Ca (-1.86%, 95% CI: -2.95; -0.76, IQR: 1.33), and Ti (-2.58%, 95% CI: -4.44; -0.68, IQR: 0.03), and FVC showed negative associations with concentrations of three elements: Si (-3.23%, 95% CI: -5.61; -0.79), Al (-3.26%, 95% CI: -5.73; -0.72), and Ca (-1.86%, 95% CI: -3.23; -0.47). In stratified analysis, Si, Al, Ca, and Ti showed associations with lung function only among truck drivers, and no significant association among office workers.

**Conclusion:**

Selected elemental components of PM_2.5_ showed effects on lung function that were not found in analyses of particle levels alone.

**Electronic supplementary material:**

The online version of this article (doi:10.1186/s12989-014-0051-7) contains supplementary material, which is available to authorized users.

## Introduction

Epidemiological studies have consistently linked short-term exposure to gaseous air pollution and ambient particulate matter (PM) with increased hospitalization and mortality from respiratory disease [1-4]. Short-term exposure to PM has been repeatedly associated with decreased forced expiratory volume in 1 second (FEV1) in human studies in Boston [5], Salt Lake City [6], Italy [7], and The Netherlands [8]. In a human controlled-exposure experiment including current smokers and ex-smokers, a 2-hour exposure to concentrated ambient fine particles was sufficient to determine a significant decrease in FEV1 compared to clean air experiments [9]. Elemental components of PM have been suggested to play critical roles in determining PM toxicity [10-12]. Previous studies have examined the association of lung function with occupational exposure to metals and other toxic components, showing decreased lung function indicators in workers exposed to aluminum (Al), iron (Fe), calcium (Ca), and silicon (Si) [13-21]. However, the contribution of elemental components of PM to respiratory effects remains largely under-investigated. Decreased forced vital capacity (FVC) and FEV_1_ have also been associated with increasing concentrations of PM_2.5_ (PM with aerodynamic diameter < 2.5 μm), diesel exhaust particles, zinc (Zn), and Fe in chronic obstructive pulmonary disease (COPD) patients who are ex-smokers or sustained quitters [7,22]. Nonetheless, the effects of elemental components of PM, among individuals not selected on the basis of respiratory disease, are under-investigated.

Beijing has been ranked among the 15 cities with the highest levels of air particles worldwide, as indicated by comparisons of ambient annual PM_10_ levels [23]. Traffic-derived PM and elemental components play important roles in air pollution in Beijing due to its very high population density, rapid increase in vehicular traffic, and energy combustion with inefficient pollution control [24]. Transported particles from industrial sources and windblown dust are also major sources of pollution [24]. Examining the effects of high levels of PM and its elemental components in a highly exposed population such as the Beijing population may provide a highly effective approach to characterizing changes in lung function.

In this study of truck drivers and office workers in Beijing, China, we evaluated the effects of traffic-related PM_2.5_ and elemental components, including elemental carbon (EC), potassium (K), sulfur (S), Fe, Si, Al, Zn, Ca, and titanium (Ti) on lung function. The two groups both had high exposure levels and were selected as samples with different occupational exposures: truck drivers are directly exposed to traffic emissions, particularly from diesel exhausts; office workers were included to represent the highly exposed residential population of Beijing. To enhance the power to identify effects on lung function, we studied each participant on two different examination days 1-2 weeks apart and assessed each participant’s exposure on the days of the exam using personal measures of PM_2.5_ and the nine elemental components.

## Material and methods

### Study population and design

The Beijing Truck Driver Air Pollution Study (BTDAS), conducted between June 15 and July 27, 2008, included 60 truck drivers and 60 indoor office workers. All study participants worked and lived in the Beijing metropolitan area and had held their current jobs for at least two years. Office workers worked in buildings that did not have central air conditioning and were given the choice to use air conditioning units available in individual office rooms during the study days. Because several of the common spaces, e.g., hallways and atria, had windows that were usually left open in the summer, office workers were exposed to high levels of indoor PM due to high penetration of ambient particles from the outdoors. In-person interviews using a detailed questionnaire were conducted to collect information on demographics, lifestyle, and other exposures. Information on time-varying factors, including tea, alcohol, and smoking, was obtained for past usual exposure as well as for each examination day. Because PM levels are highly variable on a day-to-day basis, we examined all participants on two work days separated by a 1-2 week period. Individual written informed consent was obtained from all participants prior to enrollment in the study. Institutional Review Board or equivalent approval at the participating institution (i.e., Harvard School of Public Health, Northwestern University, and Peking University Health Science Center) was obtained prior to study participant recruitment.

### Personal PM_2.5_ and elemental component measurements

We measured average personal PM_2.5_ on both examination days using personal samplers worn by the study participants during eight hours of work. The personal sampler was carried in a belt pack with the inlet clipped near the breathing zone. Each sampler setup included an Apex pump (Casella Inc., Bedford, UK), a Triplex Sharp-Cut Cyclone (BGI Inc., Waltham, MA, USA), and a 37-mm Teflon filter placed on top of a drain disc and inside a filter holder made of aluminum coated in Teflon to prevent contamination. Blank filter samples were included in the analysis and showed no contamination. The filters were kept under atmosphere-controlled conditions before and after sampling and were weighed with a microbalance (Mettler-Toledo Inc., Columbus, OH, USA). A time-weighted average of PM_2.5_ concentrations was calculated by dividing the change in filter weight before and after sampling by the volume of air sampled. We found high reproducibility of PM_2.5_ measures (r = 0.944) in replicate samples on a subset of 24 participants who wore two monitors at the same time (see Additional file 1: Figure S1). The blackness of the same filters used to measure PM_2.5_ was assessed using an EEL Model M43D smoke stain reflectometer, applying the standard black-smoke index calculations of the absorption coefficients based on reflectance [25]. We assumed a factor of 1.0 for converting the absorption coefficient to EC mass [26,27], which was then divided by the sampled air volume to calculate average EC concentration [25]. EC is a combustion by-product contained in PM_2.5_ that has been used as a surrogate measure for PM_2.5_ from gasoline- and especially diesel-powered motor vehicles [26].

Elemental components of PM_2.5_ were analyzed after the gravimetric mass measurement using a XRF PANanalytical Epsilon 5 analyzer (Almelo, The Netherlands), as described previously [28,29]. We selected from this analysis the eight elements, i.e., K, S, Fe, Si, Al, Zn, Ca, and Ti, that showed the highest reproducibility (r > 0.75) in replicate samples from the subset of 24 participants who wore two monitors at the same time (see Additional file 1: Figure S2).

### Lung function measurement

Lung function testing was performed using the EasyOne ultrasonic flow-sensing spirometer (NDD Medical Technologies, Zurich, Switzerland). The EasyOne spirometer has high validity and reproducibility [30-32]. Independent testing of the device has shown it to exceed standard thresholds for acceptability with American Thoracic Society (ATS) waveforms [30] and to have both high calibration stability [31,32] and excellent agreement with standard laboratory-based spirometers [33]. All lung function measures were performed at the end of the work day (between 4-6 pm) according to ATS guidelines [34] by a research assistant trained specifically for this study. We retained completed spirometry sessions consisting of three of six best maneuvers. Five indicators were included in our study: FEV1, FVC, FEV1/FVC ratio, forced expiratory flow 25%-75% (FEF25-75%), and peak expiratory flow (PEF).

### Statistical analysis

Standard descriptive statistics were used to present the characteristics of truck drivers and office workers. Preliminary to the analysis presented in this manuscript, we performed statistical analysis to confirm that there were no differences in lung function levels between office workers and truck drivers. We used mixed-effects regression models to test the differences in lung functions between groups and to estimate group-specific means and 95% confidence intervals (CIs). We fitted models adjusted for variables either not matched or incompletely matched by design between the two groups, i.e., age, sex, body mass index (BMI) (continuous), cigarettes smoked during the examination time (continuous), day of the week (one indicator variable per day), time spent commuting to work (continuous), work hours per week, temperature (continuous), and dew point (continuous). The mixed-effects models were based on the following equation:

1 $$ {Y}_{ij} = {\upbeta}_0 + {\upbeta}_1{\left(\mathrm{Group}\right)}_{ij} + {\upbeta}_2{X_2}_{ij}+\dots + {\upbeta}_{\mathrm{n}}{X_{\mathrm{n}}}_{ij} + {\upalpha}_1{X}_{1j}+\dots + {\upalpha}_{\mathrm{n}}{X}_{\mathrm{m}j} + {\upxi}_j+{e}_{ij} $$

where β_0_ is the overall intercept; β_1_ is the regression coefficient for the group; β_2_…β_*n*_ are the regression coefficients for the time-dependent covariates; α_1_…α_n_ are the regression coefficients for the time-independent covariates included in the multivariate models; ξ_j_ is the random effect for the subject; *j* represents the subject; *i* identifies the workday, and *e*_*ij*_ is the residual error term. Based on these models, we found no statistically significant differences in any of the lung function indicators between office workers and truck drivers (data not shown). We then used mixed-effects models adjusted for age, sex, BMI, cigarettes smoked during the examination time, day of the week, time spent commuting to work, work hours per week, temperature, and dew point to evaluate the association of personal PM_2.5_ with each of the lung function indicators (i.e., FEV1, FVC, FEF25%-75%, FEV1/FVC ratio, and PEF). The mixed-effects models used the following structure based on Model 1, with an additional exposure variable term for PM_2.5_, EC, or one of the elemental components of PM_2.5_:

2 $$ {Y}_{ij} = {\upbeta}_0 + {\upbeta}_1{\left(\mathrm{Exposure}\right)}_{ij} + {\upbeta}_2{\left(\mathrm{Group}\right)}_{ij}+{\upbeta}_3{X}_{3ij}+\dots + {\upbeta}_{\mathrm{n}}{X_{\mathrm{n}}}_{ij} + {\upalpha}_1{X}_{1j}+\dots + {\upalpha}_{\mathrm{n}}{X}_{\mathrm{m}j} + {\upxi}_j+{e}_{ij} $$

Note that PM_2.5_ was fitted as a covariate in Model 2 when evaluating the effects of personal EC or elemental components of PM_2.5_ on lung function [35]. The Benjamini and Hochberg (BH) procedure to control the false discovery rate (FDR) for multiple comparisons [36] was applied for multiple tests of significance. A two-sided BH FDR of less than 0.05 was considered noteworthy. Statistical significance for interaction was assessed by the likelihood-ratio test comparing the models with and without the interaction term [37]. All analyses were performed in SAS 9.2 (SAS Institute Inc., Cary, NC).

## Results

### Characteristics of study subjects

The characteristics of the 60 office workers and 60 truck drivers are presented in Table 1. Briefly, truck drivers were moderately but significantly older than office workers. Truck drivers had higher BMI, reported more pack-years of smoking, smoked more cigarettes during the examination time, and included a higher proportion of usual alcohol drinkers and tea consumers.

**Table 1:** **Characteristics of the study participants**

	**Office workers**	**Truck drivers**	**p-value** ^**a**^
	**(n = 60)**	**(n = 60)**	
**Sex, n (%)**			
Male	40 (66.7)	40 (66.7)	
Female	20 (33.3)	20 (33.3)	1.00
**Smoking, n (%)**			
Never smoker	35 (58.3)	34 (56.7)	
Former	2 (3.3)	2 (3.3)	
Current	23 (38.3)	24 (40.0)	1.00
**Age [years], mean (SD)**	30.3 ± 8.0	33.5 ± 5.7	0.01
**BMI [kg/m** ^**2**^ **], mean (SD)**	22.8 ± 3.4	24.3 ± 3.2	0.01
**Time spent commuting to work, mean (SD)**	6.9 ± 9.0	12.4 ± 15.2	<0.001
**Cigarettes smoked during examination time, mean (SD)** ^**b**^	0.5 ± 1.7	2.3 ± 4.2	0.003
**Work hours per week, mean ± SD**	50.6 ± 11.0	67.3 ± 14.0	<0.001
**Study day of the week, n (%)**			
Monday	16 (13.3)	19 (15.8)	
Tuesday	18 (15.0)	13 (10.8)	
Wednesday	14 (11.7)	15 (12.5)	
Thursday	15 (12.5)	20 (16.7)	
Friday	17 (14.2)	19 (15.8)	
Saturday	18 (15.0)	16 (13.3)	
Sunday	22 (18.3)	18 (15.0)	0.8^c^
**Average temperature on two study days, mean (SD)**	25.4 ± 2.5	25.3 ± 2.5	0.96^c^
**Average dew point on two study days, mean (SD)**	20.6 ± 2.1	20.6 ± 2.1	0.93^c^

^a^P-values were calculated using Student’s t-test and Fisher’s exact test for continuous and categorical variables, respectively.

^b^Only current or former smokers.

^**c**^Cumulative of the two study days. Based on 240 total observations (120 study days for office workers and 120 study days for truck drivers). P-values were obtained from mixed-effects regression models.

### Personal PM_2.5_ and elemental component levels

The levels and distribution of personal time-weighted average exposure to PM_2.5_ and nine elemental components were estimated during 8 work hours (Table 2). Average personal PM_2.5_ was 94.59 μg/m^3^ for office workers and 126.83 μg/m^3^ for truck drivers. Average personal EC was 13.01 μg/m^3^ for office workers and 17.27 μg/m^3^ for truck drivers. For the elemental components, the measured levels for truck drivers were significantly higher than those for office workers.

**Table 2:** **Level of personal PM**_**2.5**_ **, elemental carbon (EC), and elemental components of PM**_**2.5**_ **during work hours on the examination days** ^**a**^

	**Office workers**								**Truck drivers**								**Mean exposure difference (95% CI)** ^**b**^
	**(observations = 120)**								**(observations = 120)**								
	**N**	**Mean**	**SD**	**10pct**	**25pct**	**Median**	**75pct**	**90pct**	**N**	**Mean**	**SD**	**10pct**	**25pct**	**Median**	**75pct**	**90pct**	
PM_2.5_	120	94.59	64.91	22.39	48.48	86.17	126.56	183.44	119	126.83	68.82	46.26	73.86	116.78	160.49	213.94	32.24 (15.19; 49.28)
EC	118	13.01	4.04	7.11	9.99	13.16	15.85	18.35	120	17.27	6.69	8.93	12.78	16.86	21.03	26.21	4.26 (2.84; 5.68)
K	118	0.76	0.77	0.18	0.27	0.56	0.82	2.07	120	1.31	1.07	0.34	0.44	0.89	2.06	2.81	0.55 (0.31; 0.79)
S	118	6.19	5.05	0.61	1.66	5.30	8.64	13.77	120	8.43	4.92	2.30	4.87	6.98	12.47	16.14	2.24 (0.97; 3.52)
Fe	118	0.38	0.21	0.17	0.24	0.34	0.44	0.69	120	1.01	0.64	0.38	0.50	0.82	1.34	1.75	0.63 (0.51; 0.75)
Si	118	0.79	0.53	0.28	0.45	0.68	1.03	1.43	120	2.37	1.76	0.64	0.82	2.09	3.54	4.13	1.58 (1.25; 1.91)
Al	118	0.54	0.25	0.23	0.37	0.50	0.70	0.86	120	1.36	0.93	0.44	0.59	1.29	1.86	2.32	0.82 (0.65; 0.99)
Zn	118	0.15	0.17	0.02	0.05	0.08	0.22	0.37	120	0.27	0.22	0.06	0.09	0.17	0.41	0.68	0.12 (0.07; 0.17)
Ca	118	0.32	0.18	0.15	0.19	0.28	0.41	0.54	120	2.09	2.2	0.29	0.4	1.58	3.08	4.52	1.76 (1.36; 2.17)
Ti	118	0.02	0.01	0.00	0.01	0.02	0.03	0.04	120	0.06	0.04	0.02	0.03	0.05	0.08	0.10	0.04 (0.03; 0.04)

^a^Measured during the work hours of examination days using light-weight personal monitors.

^b^Mean exposure levels of truck drivers – mean exposure levels of office workers.

### Association of lung function with PM_2.5_ and elemental components

To optimize power, we conducted primary analyses on the associations between exposure measures and lung function by fitting models in all participants combined (Table 3). Secondly, we evaluated associations in office workers or truck drivers separately (Table 4). The lung function indicators were highly skewed and were log-transformed to approximate normality. In regression models, regression coefficients for log-transformed dependent variables can be rescaled to express the proportion of change associated with the exposure. Here, all results are expressed as the percent change in lung function associated with an increase equal to the interquartile range (p75 - p25) of the exposure variable. All results are covariate-adjusted and considered noteworthy at FDR < 0.05. PM_2.5_ showed no significant association with any of the five lung function indicators. Analysis of the elemental components showed that in all subjects combined, FEV1 decreased in relation to higher concentrations of four of the elements: Si (-3.07%, 95% CI: -5.00; -1.11, IQR: 1.54), Al (-2.88%, 95% CI: -4.91; -0.81, IQR: 0.86), Ca (-1.86%, 95% CI: -2.95; -0.76, IQR: 1.33), and Ti (-2.58%, 95% CI: -4.44; -0.68, IQR: 0.003), and FEV1 decreased in relation to higher concentrations of three of the elements: Si (-3.23%, 95% CI: -5.61; -0.79), Al (-3.26%, 95% CI: -5.73; -0.72), and Ca (-1.86%, 95% CI: -3.23; -0.47). The remaining three lung function indicators did not show any noteworthy associations with the elements (Table 3).

**Table 3:** **Percent change** ^**a**^ **in lung function indicators associated with an interquartile-range increase in personal levels of PM**_**2.5**_ **, elemental carbon (EC), or elemental components of PM**_**2.5**_^**b**^

		**FEV1 (observations = 237)** ^**c**^			**FVC (observations = 237)** ^**c**^			**FEV1/FVC ratio (observations = 237)** ^**c**^			**FEF 25%-75% (observations = 235)** ^**c**^			**PEF (observations = 237)** ^**c**^		
	**IQR** ^**d**^	**% change**	**(95% CI)**	**FDR** ^**e**^	**% change**	**(95% CI)**	**FDR**	**% change**	**(95% CI)**	**FDR**	**% change**	**(95% CI)**	**FDR**	**% change**	**(95% CI)**	**FDR**
PM_2.5_	83.87	1.11	(-1.31; 3.59)	0.466	0.12	(-2.79; 3.11)	0.938	1.27	(-0.91; 3.50)	0.952	8.18	(-3.01; 20.67)	0.490	3.09	(-0.47; 6.77)	0.574
EC	7.36	0.37	(-1.94; 2.74)	0.755	-1.39	(-4.14; 1.43)	0.415	0.69	(-1.47; 2.89)	0.952	7.72	(-3.09; 19.73)	0.490	0.66	(-2.65; 4.09)	0.700
K	0.91	0.51	(-1.75; 2.82)	0.734	0.72	(-2.01; 3.52)	0.680	0.51	(-1.58; 2.65)	0.952	7.07	(-3.39; 18.67)	0.490	0.66	(-2.6; 4.03)	0.700
S	6.67	1.94	(-1.07; 5.04)	0.301	1.93	(-1.82; 5.81)	0.415	0.71	(-2.23; 3.74)	0.952	9.71	(-4.89; 26.54)	0.490	1.67	(-2.68; 6.22)	0.574
Fe	0.55	-1.95	(-3.75; -0.11)	0.080	-1.76	(-3.96; 0.49)	0.252	-0.06	(-1.79; 1.70)	0.952	-2.79	(-10.77; 5.90)	0.576	-1.24	(-3.86; 1.46)	0.574
Si	1.54	-3.07	(-5.00; -1.11)	0.015	-3.23	(-5.61; -0.79)	0.043	-0.12	(-2.00; 1.79)	0.952	-3.24	(-11.96; 6.34)	0.576	-1.97	(-4.86; 1.00)	0.574
Al	0.86	-2.88	(-4.91; -0.81)	0.023	-3.26	(-5.73; -0.72)	0.043	0.30	(-1.67; 2.30)	0.952	-1.88	(-11.05; 8.25)	0.706	-1.87	(-4.86; 1.21)	0.574
Zn	0.24	1.55	(-0.44; 3.57)	0.217	1.72	(-0.75; 4.24)	0.293	0.30	(-1.71; 2.35)	0.952	5.86	(-3.78; 16.48)	0.490	1.28	(-1.58; 4.23)	0.574
Ca	1.33	-1.86	(-2.95; -0.76)	0.010	-1.86	(-3.23; -0.47)	0.043	-0.15	(-1.22; 0.94)	0.952	-2.42	(-7.50; 2.93)	0.576	-0.96	(-2.60; 0.71)	0.574
Ti	0.03	-2.58	(-4.44; -0.68)	0.023	-2.69	(-4.99; -0.34)	0.068	0.06	(-1.77; 1.91)	0.952	-3.32	(-11.68; 5.84)	0.576	-1.08	(-3.86; 1.77)	0.574

^a^Adjusted for group, age, sex, BMI, number of cigarettes smoked during examination time, day of the week, time used for commuting to work, work hours per week, temperature, and dew point values. For exposures other than PM_2.5_, PM_2.5_ was also included in the model as an independent variable.

^b^Measured during work hours of the examination days using light-weight personal monitors.

^c^Observations vary across different lung function indicators due to missing values.

^d^Percent changes and 95% confidence intervals are scaled to the interquartile range (IQR) of the exposures.

^e^FDR: Benjamini and Hochberg false discovery rate.

**Table 4:** **Percent change** ^**a**^ **in FEV1 associated with an interquartile-range increase in personal levels of PM**_**2.5**_ **, elemental carbon (EC), or elemental components of PM**_**2.5**_ **,** ^**b**^ **stratified by group**

	**Office workers (observations = 118)**				**Truck drivers (observations = 119)**			
	**IQR** ^**c**^	**% change**	**(95% CI)**	**FDR** ^**d**^	**IQR** ^**c**^	**% change**	**(95% CI)**	**FDR** ^**d**^
PM_2.5_	78.08	0.37	(-1.60; 2.38)	0.961	86.63	-0.29	(-4.18; 3.75)	0.886
EC	5.86	-0.49	(-3.29; 2.39)	0.961	8.26	-1.93	(-6.58; 2.94)	0.541
K	0.55	2.00	(0.04; 3.99)	0.500	1.61	-0.97	(-8.79; 7.52)	0.886
S	6.98	-1.52	(-5.26; 2.36)	0.961	7.61	3.65	(-3.01; 10.77)	0.490
Fe	0.19	1.20	(-0.56; 2.98)	0.940	0.84	-4.10	(-8.48; 0.49)	0.170
Si	0.58	-0.18	(-2.00; 1.68)	0.961	2.73	-8.14	(-13.77; -2.14)	0.058
Al	0.33	-0.48	(-3.17; 2.28)	0.961	1.27	-6.64	(-11.53; -1.47)	0.058
Zn	0.17	0.65	(-1.12; 2.45)	0.961	0.31	2.08	(-2.49; 6.86)	0.541
Ca	0.21	-0.12	(-2.43; 2.24)	0.961	2.68	-4.34	(-7.83; -0.73)	0.058
Ti	0.02	0.07	(-2.61; 2.82)	0.961	0.05	-5.63	(-10.07; -0.96)	0.058

^a^Adjusted for age, sex, BMI, number of cigarettes smoked during examination time, day of the week, time spent commuting to work, work hours per week, temperature, and dew point values. For exposures other than PM_2.5_, PM_2.5_ was also adjusted.

^b^Measured during work hours of the examination days using light-weight personal monitors.

^c^Percent changes and 95% confidence intervals are scaled to the interquartile range (IQR) of the exposures.

^d^FDR: Benjamini and Hochberg false discovery rate.

Stratified analyses in office workers and truck drivers showed similar associations between elemental components and FEV1 in truck drivers, but not in office workers. In addition to the four components that showed significant associations in all participants combined, Fe was also negatively associated with FEV1 among truck drivers: FEV1 was negatively associated with concentrations of Fe (-4.24%, 95% CI: -7.55; -0.8), Si (-8.13%, 95% CI: -12.3; -3.76), Al (-6.46%, 95% CI: -10.18; -2.59), Ca (-4.40%, 95% CI: -6.97; -1.77), and Ti (-5.18%, 95% CI: -8.53; -1.71). However, FEV1 was positively associated with the concentration of S (6.16%, 95% CI: 1.06; 11.51) in truck drivers (Table 4). Stratified analyses also showed similar associations between elemental components and FVC in truck drivers but not in office workers. In addition to the three components that showed significant associations in all participants combined, Ti was also marginally negatively associated with FVC among truck drivers: FVC was marginally negatively associated with concentrations of Si (-8.14%, 95% CI: -13.77; -2.14), Al (-6.46%, 95% CI: -11.53; -1.47), Ca (-4.34%, 95% CI: -7.83; -0.73), and Ti (-5.63%, 95% CI: -10.07; -0.96) (Table 5). None of the exposure variables showed associations with the other lung function indicators in analyses restricted to truck drivers or office workers (data not shown). However, although the effects of the exposure appeared stronger among drivers, formal testing showed that they were not different between the two groups at FDR < 0.05. We further examined the interaction effects between sex and exposures to PM_2.5_ and elemental components of PM on lung function by introducing an interaction term with sex into Model 2. We did not observe significant interactions between exposures and sex (see Additional file 1: Table S2).

**Table 5:** **Percent change** ^**a**^ **in FVC associated with an interquartile-range increase in personal levels of PM**_**2.5**_ **, elemental carbon (EC), or elemental components of PM**_**2.5**_^**b**^, stratified by group

	**Office workers (observations = 118)**				**Truck drivers (observations = 119)**			
	**IQR** ^**c**^	**% change**	**(95% CI)**	**FDR** ^**d**^	**IQR** ^**c**^	**% change**	**(95% CI)**	**FDR** ^**d**^
PM_2.5_	78.08	0.37	(-1.60; 2.38)	0.961	86.63	-0.29	(-4.18; 3.75)	0.886
EC	5.86	-0.49	(-3.29; 2.39)	0.961	8.26	-1.93	(-6.58; 2.94)	0.541
K	0.55	2.00	(0.04; 3.99)	0.500	1.61	-0.97	(-8.79; 7.52)	0.886
S	6.98	-1.52	(-5.26; 2.36)	0.961	7.61	3.65	(-3.01; 10.77)	0.490
Fe	0.19	1.20	(-0.56; 2.98)	0.940	0.84	-4.10	(-8.48; 0.49)	0.170
Si	0.58	-0.18	(-2.00; 1.68)	0.961	2.73	-8.14	(-13.77; -2.14)	0.058
Al	0.33	-0.48	(-3.17; 2.28)	0.961	1.27	-6.64	(-11.53; -1.47)	0.058
Zn	0.17	0.65	(-1.12; 2.45)	0.961	0.31	2.08	(-2.49; 6.86)	0.541
Ca	0.21	-0.12	(-2.43; 2.24)	0.961	2.68	-4.34	(-7.83; -0.73)	0.058
Ti	0.02	0.07	(-2.61; 2.82)	0.961	0.05	-5.63	(-10.07; -0.96)	0.058

^a^Adjusted for age, sex, BMI, number of cigarettes smoked during examination time, day of the week, time spent commuting to work, work hours per week, temperature, and dew point values. For exposures other than PM_2.5_, PM_2.5_ was also adjusted.

^b^Measured during the work hours of examination days using light-weight personal monitors.

^c^Percent changes and 95% confidence intervals are scaled to the interquartile range (IQR) of the exposures.

^d^FDR: Benjamini and Hochberg false discovery rate.

Stratified analyses in non-smokers and smokers showed stronger negative associations of FEV1 and FVC with concentrations of Fe, Si, Al, Ca, and Ti (see Additional file 1: Table S4), but no significant associations in smokers (see Additional file 1: Table S3). Stratified analysis by median BMI (23 kg/m^2^) showed strong negative associations of FEVI and FVC with concentrations of Fe, Si, Al, Ca, and Ti among participants with BMI below the median (see Additional file 1: Table S6), but no significant associations among participants with BMI above the median (see Additional file 1: Table S5).

## Discussion

In this study of truck drivers and office workers in Beijing, China, we found that higher concentrations of Si, Al, Ca, and Ti in PM_2.5_ particles were associated with decreased lung function as assessed by FEV1. Si, Al, and Ca were also negatively associated with FVC. These associations were stronger in truck drivers relative to office workers. Si, Al, Ca, and Ti are crustal elements that are common in road dust [38], which may explain why exposure to these elements was higher in truck drivers, and suggests that road dust may be implicated in these results.

Our findings are in line with previous studies on the effect of these elements on lung function in occupational settings. The observed association of FEV1 with Al is consistent with previous studies showing an association between lower FEV1 or FVC and exposure to Al in potroom workers [13], cast-house workers [14], and smelters [15], although the Al concentrations in these occupational studies exceeded the concentrations measured in the BTDAS. An experimental study suggested that Al induces oxidative and inflammatory stress, leading to damage of the lung epithelium [39]. In Al-exposed workers, Elserougy et al. reported an elevated level of C-reactive protein (CRP) [40], an inflammatory marker that has been associated with reduced FEV1 and FVC [41-45]. Although the reason underlying the inverse association is unclear, it is hypothesized that persistent systemic inflammation and pulmonary micro-filtration may result in damage to the airways, leading to a decline in FEV1 and FVC [41,46]. Si and Ca in truck drivers may represent exposure to either asphalt- or cement-paved road dusts related to traffic emissions during driving [47]. Recently, Johncy et al. found a statistically significant decrease in lung function indicators, including FEV1 and FVC, in sweepers exposed to road dust as compared to controls [48]. Therefore, road dust containing a wide range of organic compounds may have an effect on lung function. Cement factory workers are also exposed to a mixture of components, including Ca, Si, and Al, which have been linked to respiratory function deficits [49]. Silicon dioxide, an ubiquitous substance in cement factories, can be inhaled and become embedded deep into the alveolar sacs to start an inflammatory reaction releasing chemokine [50]. Such persistent chronic irritation caused by cement could induce inflammatory responses, which have been repeatedly associated with reduced lung function [51,52]. Several human studies have found decreased lung function in cement factory workers [17-21].

Our study had the advantage of having personal measures of PM_2.5_, EC, and elemental components of PM_2.5_. All participants were evaluated with standard validated protocols for PM_2.5_ assessment and measurement of lung function indicators. We conducted technical validation of personal PM_2.5_ (r = 0.944) and eight elemental component (r > 0.75) measures and observed high measurement reproducibility. By measuring EC, a tracer of traffic particles, as well as by evaluating a group, i.e., truck drivers with direct exposure to traffic, we had the opportunity to distinguish the effects of traffic pollution from those of the general levels of ambient PM_2.5_ in Beijing.

We also recognize that our study is subject to a number of limitations. For example, we cannot exclude that other unexamined exposures may have also affected FEV1 or FVC levels. We also cannot exclude false negative findings or chance findings due to the relatively small sample size, such as the positive association between S and FEV1 in truck drivers; however, all our findings were noteworthy using a stringent cutoff of 0.05 for FDR, limiting the chance of false positive results. Because the four elemental components with significant associations with lung function were highly correlated with each other (r > 0.88), we may not be able to separate the individual effects of each specific element. Moreover, whether our findings can be extended to the winter season in Beijing in this group of healthy individuals remains to be determined, as our study was conducted only during the warm season. In a study of 76 COPD patients living in East London, Donaldson et al. showed that FEV1 and FVC fell markedly between the warmest and the coolest week of the study, suggesting a temperature-related reduction in lung function [53]. Differences in sources of air pollutants, PM chemical composition, and daily behavior by season may all lead to seasonal variation in the effects of PM on lung function [54]. We could not examine the temporal scale of effects, i.e., the latency time from exposure to respiratory effects in our study, which was designed to investigate short-term exposure. Further studies are needed to generalize the results of our study to other settings with lower or long-term exposure, different climates, and/or different sources of pollution. Various cytokines, such as IL2, IL4, IL6, IL8, and TNFα, and other inflammatory markers, such as intercellular adhesion molecule (ICAM) 1, soluble P-selectin, and CRP, have previously been associated with metal exposure or respiratory diseases [40,55,56]. Therefore, further studies examining these markers are needed to better clarify the role of inflammation in the relation of lung function to PM and elemental component exposure.

Overall, our investigation provides evidence that exposure to elemental components of PM_2.5_, such as Si, Al, Ca, and Ti, is associated with reduced lung function. The lack of associations with personal PM_2.5_ and EC measured during work hours indicates that measured elemental components of PM_2.5_ may provide valuable information to determine the effects of PM_2.5_ on lung function. Our results further support the urgent implementation of exposure reduction measures in the Beijing metropolitan area as well as in areas with similarly high levels of potentially toxic components worldwide.

## Additional file

Additional file 1:
**Figure S1.** Measures of PM_2.5_ from two independent personal monitors worn at the same time by a subset of 12 study subjects to test the accuracy of the measurements. **Figure S2.** Measures of top 8 inhaled toxic metals from two independent personal monitors. **Table S1.** Pearson correlation coefficients table for the eight selected metals, PM_2.5_, and EC. **Table S2.** Interactions between air particle exposures and sex on lung function. **Table S3.** Percent change in lung function indicators associated with an interquartile-range increase in personal levels of PM_2.5_, elemental carbon (EC), or elemental components of PM_2.5_ in smokers. **Table S4.** Percent change in lung function indicators associated with an interquartile-range increase in personal levels of PM_2.5_, elemental carbon (EC), or elemental components of PM_2.5_ in non-smokers. **Table S5.** Percent change in lung function indicators associated with an interquartile-range increase in personal levels of PM_2.5_, elemental carbon (EC), or elemental components of PM_2.5_ in participants with high BMI (above the median). **Table S6.** Percent change in lung function indicators associated with an interquartile-range increase in personal levels of PM_2.5_, elemental carbon (EC), or elemental components of PM_2.5_ in participants with low BMI (below the median).

## Abbreviations

Al: Aluminum
BH: Benjamini and Hochberg
BMI: body mass index
BTDAS: Beijing Truck Driver Air Pollution Study
Ca: calcium
CI: confidence interval
COPD: chronic obstructive pulmonary disease
CRP: C-reactive protein
EC: elemental carbon
FDR: false discovery rate
FEV1: forced expiratory volume in 1 second
FVC: forced vital capacity
Fe: iron
FEF25-75%: forced expiratory flow 25%-75%
ICAM: intercellular adhesion molecule
IQR: inter-quartile range
K: potassium
PEF: peak expiratory flow
PM: particulate matter
PM_2.5_: particulate matter ≤ 2.5 μm
S: sulfur
SD: standard deviation
Si: silicon
Ti: titanium
Zn: zinc
