# Supplementary material for: Air pollution exposure and lung function in highly exposed subjects in Beijing, China: a repeated-measure study
Source: Part Fibre Toxicol. 2014 Oct 2;11:51. doi: 10.1186/s12989-014-0051-7 (PMC4192276; doi:10.1186/s12989-014-0051-7)
Supplement: Additional file 1: — Figure S1. Measures of PM2.5 from two independent personal monitors worn at the same time by a subset of 12 study subjects to test the accuracy of the measurements. Figure S2. Measures of top 8 inhaled toxic metals from two independent personal monitors. Table S1. Pearson correlation coefficients table for the eight selected metals, PM2.5, and EC. Table S2. Interactions between air particle exposures and sex on lung function. Table S3. Percent change in lung function indicators associated with an interquartile-range increase in personal levels of PM2.5, elemental carbon (EC), or elemental components of PM2.5 in smokers. Table S4. Percent change in lung function indicators associated with an interquartile-range increase in personal levels of PM2.5, elemental carbon (EC), or elemental components of PM2.5 in non-smokers. Table S5. Percent change in lung function indicators associated with an interquartile-range increase in personal levels of PM2.5, elemental carbon (EC), or elemental components of PM2.5 in participants with high BMI (above the median). Table S6. Percent change in lung function indicators associated with an interquartile-range increase in personal levels of PM2.5, elemental carbon (EC), or elemental components of PM2.5 in participants with low BMI (below the median). [file 12989_2014_51_MOESM1_ESM.doc]

**Additional Files**

Additional File 1

**
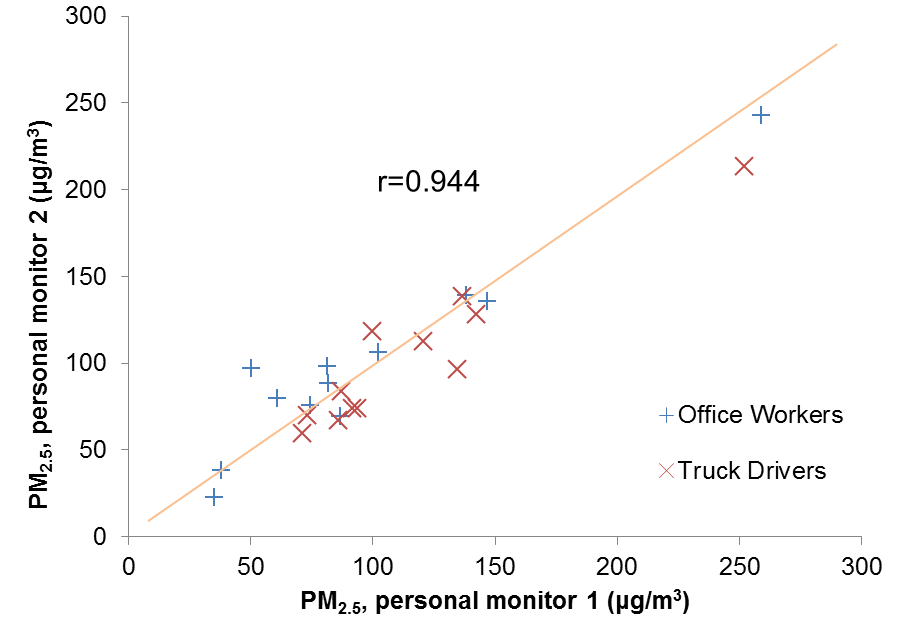
**

**Figure S1.** Measures of PM_2.5_ from two independent personal monitors worn at the same time by a subset of 12 study subjects to test the accuracy of the measurements. The scatter plot shows the high correlation (Pearson’s *r*=0.944) between monitor 1 and monitor 2.


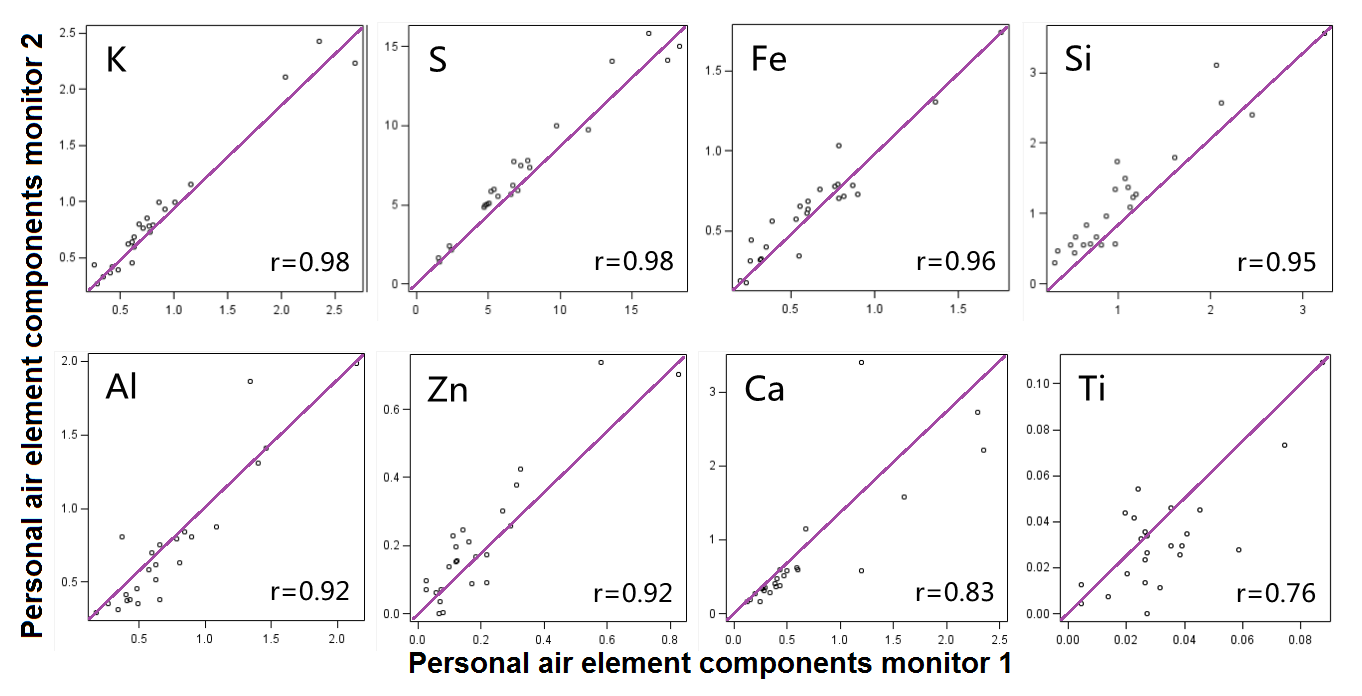


**Figure S2.** Measures of elemental components from two independent personal monitors worn at the same time by a subset of 12 study subjects to test the accuracy of the measurements. The figure show scatterplots for the eight elements with the highest accuracy, based on Pearson’s correlation coefficients (*r*).

**Table S1**. Pearson correlation coefficients table for the eight selected elemental components, PM_2.5_ and elemental carbon (EC).

|  | **K** | **S** | **Fe** | **Si** | **Al** | **Zn** | **Ca** | **Ti** | **PM_2.5_** |
| --- | --- | --- | --- | --- | --- | --- | --- | --- | --- |
| **S** | 0.78 |  |  |  |  |  |  |  |  |
|  | P<0.001 |  |  |  |  |  |  |  |  |
| **Fe** | 0.74 | 0.55 |  |  |  |  |  |  |  |
|  | P<0.001 | P<0.001 |  |  |  |  |  |  |  |
| **Si** | 0.65 | 0.48 | 0.89 |  |  |  |  |  |  |
|  | P<0.001 | P<0.001 | P<0.001 |  |  |  |  |  |  |
| **Al** | 0.69 | 0.54 | 0.90 | **0.96** |  |  |  |  |  |
|  | P<0.001 | P<0.001 | P<0.001 | P<0.001 |  |  |  |  |  |
| **Zn** | 0.70 | 0.71 | 0.63 | 0.52 | 0.50 |  |  |  |  |
|  | P<0.001 | P<0.001 | P<0.001 | P<0.001 | P<0.001 |  |  |  |  |
| **Ca** | 0.57 | 0.38 | 0.82 | **0.93** | **0.93** | 0.34 |  |  |  |
|  | P<0.001 | P<0.001 | P<0.001 | P<0.001 | P<0.001 | P<0.001 |  |  |  |
| **Ti** | 0.65 | 0.50 | 0.88 | **0.91** | **0.92** | 0.49 | **0.88** |  |  |
|  | P<0.001 | P<0.001 | P<0.001 | P<0.001 | P<0.001 | P<0.001 | P<0.001 |  |  |
| **PM_2.5_** | 0.83 | 0.79 | 0.62 | 0.63 | 0.67 | 0.55 | 0.57 | 0.64 |  |
|  | P<0.001 | P<0.001 | P<0.001 | P<0.001 | P<0.001 | P<0.001 | P<0.001 | P<0.001 |  |
| **EC** | 0.58 | 0.46 | 0.57 | 0.55 | 0.59 | 0.38 | 0.52 | 0.52 | 0.54 |
|  | P<0.001 | P<0.001 | P<0.001 | P<0.001 | P<0.001 | P<0.001 | P<0.001 | P<0.001 | P<0.001 |

**Table S2.** Interactions between personal levels of PM_2.5_, elemental carbon (EC), or elemental components of PM_2.5_ exposures and sex on lung functions

| **Exposure^a^** | **P-value for interaction^b^** | | | | |
| --- | --- | --- | --- | --- | --- |
|  | **FEV1** | **FVC** | **FEV1/FVC ratio** | **FEF25%-75%** | **PEF** |
| PM_2.5_ | 0.691 | 0.639 | 0.101 | 0.025 | 0.364 |
| EC | 0.608 | 0.375 | 0.051 | 0.118 | 0.898 |
| K | 0.468 | 0.980 | 0.786 | 0.372 | 0.568 |
| S | 0.106 | 0.629 | 0.305 | 0.173 | 0.308 |
| Fe | 0.369 | 0.338 | 0.367 | 0.302 | 0.313 |
| Si | 0.873 | 0.922 | 0.910 | 0.728 | 0.471 |
| Al | 0.357 | 0.900 | 0.598 | 0.828 | 0.449 |
| Zn | 0.140 | 0.191 | 0.677 | 0.973 | 0.584 |
| Ca | 0.345 | 0.151 | 0.161 | 0.098 | 0.382 |
| Ti | 0.562 | 0.395 | 0.435 | 0.438 | 0.245 |

^a^ Measured during the work hours of examination days using light-weight personal monitors.

^b^ Based on mixed-effects model (model 2, as described in the statistical methods), an interaction term of sex and exposure was added. The results were adjusted for group, age, sex, BMI, number of cigarettes smoked during examination time, day of the week, time used for commuting to work, work hours per week, temperature, and dew point values. For exposures other than PM_2.5_, PM_2.5_ was also adjusted.

**Table S3.** Percent changea in lung function indicators associated with an interquartile-range increase in personal levels of PM2.5, elemental carbon (EC), or elemental components of PM2.5b in smokers

|  |  |  |  |  | **FEV1 (obs=100)**^c^ | | |  | **FVC (obs=100)**^c^ | | |  | **FEV1/FVC ratio (obs=100)**^c^ | | |
| --- | --- | --- | --- | --- | --- | --- | --- | --- | --- | --- | --- | --- | --- | --- | --- |
|  |  |  | **IQR^d^** |  | **% change** | **95% CI** | **FDR^e^** |  | **% change** | **95% CI** | **FDR^e^** |  | **% change** | **95% CI** | **FDR^e^** |
|  | PM_2.5_ |  | 92.68 |  | 0.51 | (-1.37;2.43) | 0.749 |  | 0.36 | (-3.88;4.79) | 0.953 |  | 0.57 | (-2.89;4.15) | 0.836 |
|  | EC |  | 8.19 |  | -0.85 | (-2.99;1.33) | 0.749 |  | -4.08 | (-8.48;0.52) | 0.880 |  | 1.01 | (-2.94;5.12) | 0.781 |
|  | K |  | 1.60 |  | -1.13 | (-4.66;2.54) | 0.749 |  | -3.23 | (-10.30;4.40) | 0.953 |  | 3.82 | (-2.38;10.41) | 0.781 |
|  | S |  | 7.94 |  | -0.38 | (-3.21;2.54) | 0.888 |  | -2.01 | (-8.35;4.77) | 0.953 |  | 1.57 | (-3.99;7.46) | 0.781 |
|  | Fe |  | 0.85 |  | 0.72 | (-1.50;2.98) | 0.749 |  | -0.60 | (-5.52;4.58) | 0.953 |  | -1.3 | (-5.42;3.00) | 0.781 |
|  | Si |  | 2.13 |  | 0.95 | (-1.32;3.28) | 0.749 |  | -0.26 | (-5.31;5.07) | 0.953 |  | -2.15 | (-6.18;2.05) | 0.781 |
|  | Al |  | 1.11 |  | 0.97 | (-1.30;3.29) | 0.749 |  | -0.15 | (-5.14;5.09) | 0.953 |  | -1.62 | (-5.62;2.55) | 0.781 |
|  | Zn |  | 0.30 |  | -1.02 | (-2.84;0.83) | 0.749 |  | -0.48 | (-4.74;3.98) | 0.953 |  | 0.05 | (-3.70;3.96) | 0.978 |
|  | Ca |  | 2.03 |  | 0.40 | (-0.98;1.79) | 0.749 |  | 0.33 | (-2.83;3.58) | 0.953 |  | -1.26 | (-3.77;1.33) | 0.781 |
|  | Ti |  | 0.05 |  | 0.14 | (-2.04;2.36) | 0.902 |  | -1.15 | (-5.93;3.88) | 0.953 |  | -1.27 | (-5.27;2.89) | 0.781 |

^a^ Adjusted for group, age, sex, BMI, number of cigarettes smoked during examination time, day of the week, time spent commuting to work, work hours per week, temperature and dew-point values. For exposures other than PM_2.5_, PM_2.5_ was also included in the model as an independent variable.

^b^ Measured during work hours of the examination days using light-weight personal monitors.

^c^ Observations vary across different lung function indicators due to missing values.

^d^ Percent changes and 95% confidence intervals are scaled to the interquartile range (IQR) of the exposures.

^e^ FDR: Benjamini and Hochberg false discovery rate

(**Table S3**. continued)

|  |  |  |  |  | **FEF 25%-75% (obs=99)**^c^ | | |  | **PEF (obs=100)**^c^ | | |
| --- | --- | --- | --- | --- | --- | --- | --- | --- | --- | --- | --- |
|  |  |  | **IQR^d^** |  | **% change** | **95% CI** | **FDR^e^** |  | **% change** | **95% CI** | **FDR^e^** |
|  | PM_2.5_ |  | 92.68 |  | 9.51 | (-10.31;33.71) | 0.518 |  | -0.21 | (-3.45;3.13) | 0.980 |
|  | EC |  | 8.19 |  | 9.50 | (-11.75;35.86) | 0.518 |  | -0.76 | (-4.34;2.97) | 0.980 |
|  | K |  | 1.60 |  | 23.06 | (-12.17;72.42) | 0.518 |  | -1.21 | (-7.14;5.11) | 0.980 |
|  | S |  | 7.94 |  | 12.34 | (-17.69;53.32) | 0.520 |  | -0.07 | (-4.93;5.05) | 0.980 |
|  | Fe |  | 0.85 |  | -11.82 | (-29.70;10.61) | 0.518 |  | -1.81 | (-5.48;2.01) | 0.980 |
|  | Si |  | 2.13 |  | -11.94 | (-30.29;11.25) | 0.518 |  | -1.60 | (-5.35;2.31) | 0.980 |
|  | Al |  | 1.11 |  | -9.37 | (-27.92;13.95) | 0.518 |  | -1.20 | (-4.97;2.72) | 0.980 |
|  | Zn |  | 0.30 |  | 5.37 | (-13.98;29.07) | 0.616 |  | -1.58 | (-4.66;1.61) | 0.980 |
|  | Ca |  | 2.03 |  | -6.48 | (-18.98;7.95) | 0.518 |  | 0.08 | (-2.33;2.54) | 0.980 |
|  | Ti |  | 0.05 |  | -10.67 | (-28.72;11.94) | 0.518 |  | -0.34 | (-4.03;3.49) | 0.980 |

**Table S4.** Percent changea in lung function indicators associated with an interquartile-range increase in personal levels of PM2.5, elemental carbon (EC), or elemental components of PM2.5b in non-smokers

|  |  |  |  |  | **FEV1 (obs=137)**^c^ | | |  | **FVC (obs=137)**^c^ | | |  | **FEV1/FVC ratio (obs=137)**^c^ | | |
| --- | --- | --- | --- | --- | --- | --- | --- | --- | --- | --- | --- | --- | --- | --- | --- |
|  |  |  | **IQR^d^** |  | **% change** | **95% CI** | **FDR^e^** |  | **% change** | **95% CI** | **FDR^e^** |  | **% change** | **95% CI** | **FDR^e^** |
|  | PM_2.5_ |  | 78.08 |  | 1.97 | (-2.4;6.54) | 0.428 |  | -2.70 | (-6.69;1.47) | 0.343 |  | 4.65 | (1.56;7.83) | 0.040 |
|  | EC |  | 5.86 |  | 1.92 | (-1.52;5.47) | 0.351 |  | 1.35 | (-1.87;4.66) | 0.466 |  | 0.51 | (-1.86;2.93) | 0.679 |
|  | K |  | 0.55 |  | 0.39 | (-2.14;3.00) | 0.764 |  | -0.15 | (-2.54;2.29) | 0.902 |  | 0.93 | (-0.84;2.73) | 0.623 |
|  | S |  | 6.98 |  | 3.58 | (-2.05;9.54) | 0.317 |  | 3.23 | (-2.24;9.00) | 0.367 |  | 0.88 | (-3.18;5.10) | 0.679 |
|  | Fe |  | 0.19 |  | -3.59 | (-6.07;-1.04) | 0.016 |  | -4.17 | (-6.53;-1.75) | 0.002 |  | 1.01 | (-0.91;2.96) | 0.623 |
|  | Si |  | 0.58 |  | -4.75 | (-6.71;-2.74) | <0.001 |  | -5.01 | (-6.91;-3.06) | <0.001 |  | 0.74 | (-0.92;2.44) | 0.623 |
|  | Al |  | 0.33 |  | -3.64 | (-5.50;-1.74) | <0.001 |  | -4.00 | (-5.80;-2.18) | <0.001 |  | 0.68 | (-0.84;2.22) | 0.623 |
|  | Zn |  | 0.17 |  | 3.78 | (0.11;7.58) | 0.080 |  | 1.55 | (-1.93;5.16) | 0.466 |  | 2.59 | (-0.19;5.44) | 0.365 |
|  | Ca |  | 0.21 |  | -0.85 | (-1.24;-0.45) | <0.001 |  | -0.90 | (-1.29;-0.51) | <0.001 |  | 0.10 | (-0.23;0.42) | 0.679 |
|  | Ti |  | 0.02 |  | -5.03 | (-7.55;-2.45) | <0.001 |  | -5.38 | (-7.81;-2.89) | <0.001 |  | 0.84 | (-1.24;2.96) | 0.623 |

^a^ Adjusted for group, age, sex, BMI, number of cigarettes smoked during examination time, day of the week, time used for commuting to work, work hours per week, temperature and dew point values. For exposures other than PM_2.5_, PM_2.5_ was also included in the model as an independent variable.

^b^ Measured during work hours of the examination days using light-weight personal monitors.

^c^ Observations vary across different lung function indicators due to missing values.

^d^ Percent changes and 95% confidence intervals are scaled to the interquartile-range (IQR) of the exposures.

^e^ FDR: Benjamini and Hochberg false discovery rate

(**Table S4**. continued)

|  |  |  |  |  | **FEF 25%-75% (obs=136)**^c^ | | |  | **PEF (obs=137)**^c^ | | |
| --- | --- | --- | --- | --- | --- | --- | --- | --- | --- | --- | --- |
|  |  |  | **IQR^d^** |  | **% change** | **95% CI** | **FDR^e^** |  | **% change** | **95% CI** | **FDR^e^** |
|  | PM_2.5_ |  | 83.87 |  | 15.98 | (1.92;31.97) | 0.255 |  | 7.41 | (1.28;13.92) | 0.200 |
|  | EC |  | 7.36 |  | 6.09 | (-4.11;17.38) | 0.640 |  | 1.77 | (-2.86;6.63) | 0.661 |
|  | K |  | 0.91 |  | 5.30 | (-2.36;13.57) | 0.617 |  | 0.59 | (-2.8;4.11) | 0.737 |
|  | S |  | 6.67 |  | 6.71 | (-9.76;26.18) | 0.759 |  | 1.98 | (-5.36;9.89) | 0.676 |
|  | Fe |  | 0.55 |  | 1.04 | (-7.04;9.82) | 0.809 |  | -1.09 | (-4.62;2.58) | 0.676 |
|  | Si |  | 1.54 |  | -2.00 | (-8.82;5.34) | 0.759 |  | -2.15 | (-5.19;0.99) | 0.452 |
|  | Al |  | 0.86 |  | -1.35 | (-7.55;5.27) | 0.759 |  | -1.58 | (-4.30;1.21) | 0.452 |
|  | Zn |  | 0.24 |  | 11.52 | (0.18;24.13) | 0.255 |  | 3.43 | (-1.52;8.63) | 0.452 |
|  | Ca |  | 1.33 |  | -0.38 | (-1.73;0.99) | 0.759 |  | -0.41 | (-0.99;0.17) | 0.452 |
|  | Ti |  | 0.03 |  | -2.09 | (-10.41;6.99) | 0.759 |  | -2.17 | (-5.90;1.70) | 0.452 |

**Table S5.** Percent changea in lung function indicators associated with an interquartile-range increase in personal levels of PM2.5, elemental carbon (EC), or elemental components of PM2.5b in participants with high BMI (above the median)

|  |  |  |  |  | **FEV1 (obs=120)** | | |  | **FVC (obs=120)** | | |  | **FEV1/FVC ratio (obs=120)** | | |
| --- | --- | --- | --- | --- | --- | --- | --- | --- | --- | --- | --- | --- | --- | --- | --- |
|  |  |  | **IQR^c^** |  | **% change** | **95% CI** | **FDR^e^** |  | **% change** | **95% CI** | **FDR^e^** |  | **% change** | **95% CI** | **FDR^e^** |
|  | PM_2.5_ |  | 87.85 |  | 0.89 | (-1.98;3.84) | 0.975 |  | 0.16 | (-3.99;4.49) | 0.941 |  | 1.57 | (-1.51;4.75) | 0.326 |
|  | EC |  | 7.14 |  | -0.49 | (-2.92;2.00) | 0.975 |  | -2.84 | (-6.39;0.84) | 0.877 |  | 2.16 | (-0.75;5.15) | 0.238 |
|  | K |  | 1.30 |  | 2.06 | (-1.49;5.74) | 0.975 |  | -0.31 | (-5.44;5.10) | 0.941 |  | 3.59 | (-0.47;7.81) | 0.238 |
|  | S |  | 7.80 |  | 0.06 | (-3.75;4.02) | 0.975 |  | -2.12 | (-7.89;4.02) | 0.877 |  | 3.50 | (-1.30;8.52) | 0.238 |
|  | Fe |  | 0.79 |  | 0.16 | (-2.94;3.35) | 0.975 |  | -2.09 | (-6.54;2.57) | 0.877 |  | 2.65 | (-1.00;6.44) | 0.238 |
|  | Si |  | 2.04 |  | 1.35 | (-3.08;5.98) | 0.975 |  | -3.52 | (-9.59;2.95) | 0.877 |  | 4.21 | (-0.91;9.59) | 0.238 |
|  | Al |  | 1.10 |  | 2.36 | (-2.35;7.30) | 0.975 |  | -3.20 | (-9.54;3.58) | 0.877 |  | 6.10 | (0.82;11.65) | 0.238 |
|  | Zn |  | 0.32 |  | 0.06 | (-2.78;2.99) | 0.975 |  | -0.78 | (-5.20;3.85) | 0.922 |  | 2.09 | (-1.64;5.96) | 0.312 |
|  | Ca |  | 1.92 |  | 1.09 | (-1.85;4.11) | 0.975 |  | -1.45 | (-5.77;3.07) | 0.877 |  | 2.49 | (-0.98;6.08) | 0.238 |
|  | Ti |  | 0.05 |  | 0.99 | (-2.60;4.70) | 0.975 |  | -1.17 | (-6.51;4.47) | 0.922 |  | 2.54 | (-1.80;7.06) | 0.312 |

^a^ Adjusted for group, age, sex, number of cigarettes smoked during examination time, day of the week, time spent commuting to work, work hours per week, temperature and dew-point values. For exposures other than PM_2.5_, PM_2.5_ was also included in the model as an independent variable.

^b^ Measured during work hours of the examination days using light-weight personal monitors.

^c^ Percent changes and 95% confidence intervals are scaled to the interquartile-range (IQR) of the exposures.

^e^ FDR: Benjamini and Hochberg false discovery rate

(**Table S5**. continued)

|  |  |  |  |  | **FEF 25%-75% (obs=120)** | | |  | **PEF (obs=120)** | | |
| --- | --- | --- | --- | --- | --- | --- | --- | --- | --- | --- | --- |
|  |  |  | **IQR^c^** |  | **% change** | **95% CI** | **FDR^e^** |  | **% change** | **95% CI** | **FDR^e^** |
|  | PM_2.5_ |  | 87.85 |  | 13.45 | (-6.11;37.09) | 0.281 |  | 1.87 | (-2.24;6.15) | 0.545 |
|  | EC |  | 7.14 |  | 19.82 | (1.67;41.21) | 0.167 |  | -0.85 | (-4.33;2.76) | 0.763 |
|  | K |  | 1.30 |  | 24.86 | (-1.05;57.56) | 0.167 |  | 2.74 | (-2.46;8.23) | 0.545 |
|  | S |  | 7.80 |  | 29.64 | (-1.02;69.81) | 0.167 |  | 0.88 | (-4.69;6.78) | 0.763 |
|  | Fe |  | 0.79 |  | 11.97 | (-9.20;38.08) | 0.313 |  | 2.38 | (-2.19;7.17) | 0.545 |
|  | Si |  | 2.04 |  | 28.93 | (-3.66;72.54) | 0.186 |  | 4.43 | (-1.98;11.26) | 0.545 |
|  | Al |  | 1.10 |  | 38.56 | (2.57;87.17) | 0.167 |  | 3.90 | (-2.89;11.16) | 0.545 |
|  | Zn |  | 0.32 |  | 17.06 | (-4.64;43.7) | 0.230 |  | 0.70 | (-3.50;5.07) | 0.763 |
|  | Ca |  | 1.92 |  | 11.11 | (-9.27;36.06) | 0.313 |  | 4.03 | (-0.25;8.48) | 0.545 |
|  | Ti |  | 0.05 |  | 15.40 | (-10.04;48.03) | 0.313 |  | 2.57 | (-2.68;8.10) | 0.545 |

**Table S6.** Percent change a in lung function indicators associated with an interquartile-range increase in personal levels of PM2.5, elemental carbon (EC), or elemental components of PM2.5 b in participants with low BMI (below the median)

|  |  |  |  |  | **FEV1 (obs=117)**^c^ | | |  | **FVC (obs=117)**^c^ | | |  | **FEV1/FVC ratio (obs=117)**^c^ | | |
| --- | --- | --- | --- | --- | --- | --- | --- | --- | --- | --- | --- | --- | --- | --- | --- |
|  |  |  | **IQR^d^** |  | **% change** | **95% CI** | **FDR^e^** |  | **% change** | **95% CI** | **FDR^e^** |  | **% change** | **95% CI** | **FDR^e^** |
|  | PM_2.5_ |  | 75.29 |  | 1.29 | (-2.70;5.45) | 0.543 |  | -1.48 | (-5.20;2.40) | 0.566 |  | 2.23 | (-0.80;5.35) | 0.550 |
|  | EC |  | 5.85 |  | 1.18 | (-2.56;5.07) | 0.543 |  | 0.74 | (-2.78;4.40) | 0.705 |  | -0.33 | (-3.01;2.42) | 0.812 |
|  | K |  | 0.63 |  | -1.44 | (-5.51;2.82) | 0.543 |  | -0.78 | (-4.71;3.31) | 0.705 |  | -0.43 | (-3.34;2.55) | 0.812 |
|  | S |  | 6.31 |  | 4.66 | (-1.84;11.59) | 0.242 |  | 2.58 | (-3.66;9.23) | 0.566 |  | 1.56 | (-3.40;6.78) | 0.684 |
|  | Fe |  | 0.37 |  | -3.99 | (-6.14;-1.80) | 0.002 |  | -3.01 | (-5.20;-0.78) | 0.022 |  | -1.08 | (-2.88;0.76) | 0.550 |
|  | Si |  | 0.87 |  | -2.97 | (-4.44;-1.48) | 0.001 |  | -2.31 | (-3.81;-0.79) | 0.011 |  | -0.85 | (-2.12;0.42) | 0.550 |
|  | Al |  | 0.45 |  | -2.80 | (-4.23;-1.36) | 0.001 |  | -2.41 | (-3.82;-0.97) | 0.011 |  | -0.58 | (-1.83;0.68) | 0.550 |
|  | Zn |  | 0.18 |  | 3.75 | (-0.25;7.91) | 0.119 |  | 2.19 | (-1.68;6.21) | 0.461 |  | 1.41 | (-1.72;4.64) | 0.550 |
|  | Ca |  | 0.37 |  | -0.85 | (-1.25;-0.45) | 0.001 |  | -0.63 | (-1.05;-0.22) | 0.011 |  | -0.25 | (-0.61;0.10) | 0.550 |
|  | Ti |  | 0.02 |  | -3.36 | (-5.21;-1.48) | 0.002 |  | -2.81 | (-4.63;-0.96) | 0.011 |  | -0.77 | (-2.33;0.82) | 0.550 |

^a^ Adjusted for group, age, sex, number of cigarettes smoked during examination time, day of the week, time spent commuting to work, work hours per week, temperature and dew-point values. For exposures other than PM_2.5_, PM_2.5_ was also included in the model as an independent variable.

^b^ Measured during work hours of the examination days using light-weight personal monitors.

^c^ Observations vary across different lung function indicators due to missing values.

^d^ Percent changes and 95% confidence intervals are scaled to the interquartile-range (IQR) of the exposures.

^e^ FDR: Benjamini and Hochberg false discovery rate

(**Table S6**. continued)

|  |  |  |  |  | **FEF 25%-75% (obs=115)**^c^ | | |  | **PEF (obs=117)**^c^ | | |
| --- | --- | --- | --- | --- | --- | --- | --- | --- | --- | --- | --- |
|  |  |  | **IQR^d^** |  | **% change** | **95% CI** | **FDR^e^** |  | **% change** | **95% CI** | **FDR^e^** |
|  | PM_2.5_ |  | 87.85 |  | 10.36 | (-0.07;21.88) | 0.095 |  | 4.12 | (-1.90;10.51) | 0.271 |
|  | EC |  | 7.14 |  | 0.82 | (-8.23;10.75) | 0.866 |  | 1.08 | (-4.38;6.84) | 0.785 |
|  | K |  | 1.30 |  | -1.39 | (-11.30;9.62) | 0.866 |  | 0.71 | (-5.27;7.07) | 0.822 |
|  | S |  | 7.80 |  | 11.89 | (-4.56;31.17) | 0.215 |  | 5.96 | (-3.74;16.65) | 0.303 |
|  | Fe |  | 0.79 |  | -7.37 | (-12.70;-1.73) | 0.044 |  | -4.04 | (-7.28;-0.68) | 0.071 |
|  | Si |  | 2.04 |  | -5.56 | (-9.38;-1.57) | 0.044 |  | -3.27 | (-5.47;-1.02) | 0.063 |
|  | Al |  | 1.10 |  | -4.92 | (-8.64;-1.04) | 0.044 |  | -2.61 | (-4.82;-0.34) | 0.071 |
|  | Zn |  | 0.32 |  | 7.39 | (-2.90;18.77) | 0.215 |  | 5.05 | (-0.94;11.41) | 0.202 |
|  | Ca |  | 1.92 |  | -1.38 | (-2.47;-0.28) | 0.044 |  | -0.76 | (-1.40;-0.12) | 0.071 |
|  | Ti |  | 0.05 |  | -5.90 | (-10.68;-0.85) | 0.053 |  | -2.40 | (-5.32;0.60) | 0.202 |
